# A novel combination of mutations leading to congenital ichthyosis and ichthyosis vulgaris

**DOI:** 10.1002/ccr3.7910

**Published:** 2023-09-19

**Authors:** Zackary Shearer, Gwenevere White, John Zachary Steed, Carla Brown, Tara Venable, Megan Baber

**Affiliations:** ^1^ University of Arkansas for Medical Sciences Little Rock Arkansas USA

**Keywords:** dermatology, genetics, pediatrics and adolescent medicine

## Abstract

Coexistence of TGM1 and FLG mutations in a newborn with congenital ichthyosis is not well described in the literature. Early genetic testing and counseling are crucial for accurate diagnosis and appropriate management. Further exploration of associated problems, including hearing loss and developmental delay, is warranted in patients with these mutations.

## Graphical abstract

Infant with collodion membrane just after birth.

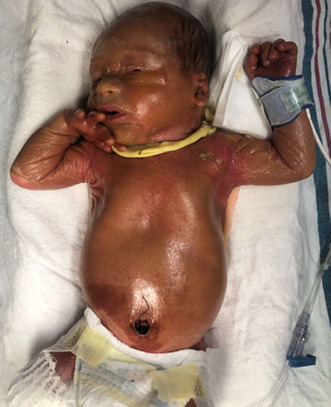

## 1 INTRODUCTION

An infant presented to the neonatal intensive care unit (NICU) with collodion membrane. Work‐up led to the discovery of a unique combination of genetic mutations (FLG and TGM 1), contributing to this condition. Conservative management with bland emollients and humidity were used for this infant. This case report describes a rare combination of mutations leading to congenital ichthyosis, along with other clinical findings, including hearing loss and developmental delay, in the affected patient.

## 2 CASE HISTORY/EXAMINATION

**FIGURE 1:**
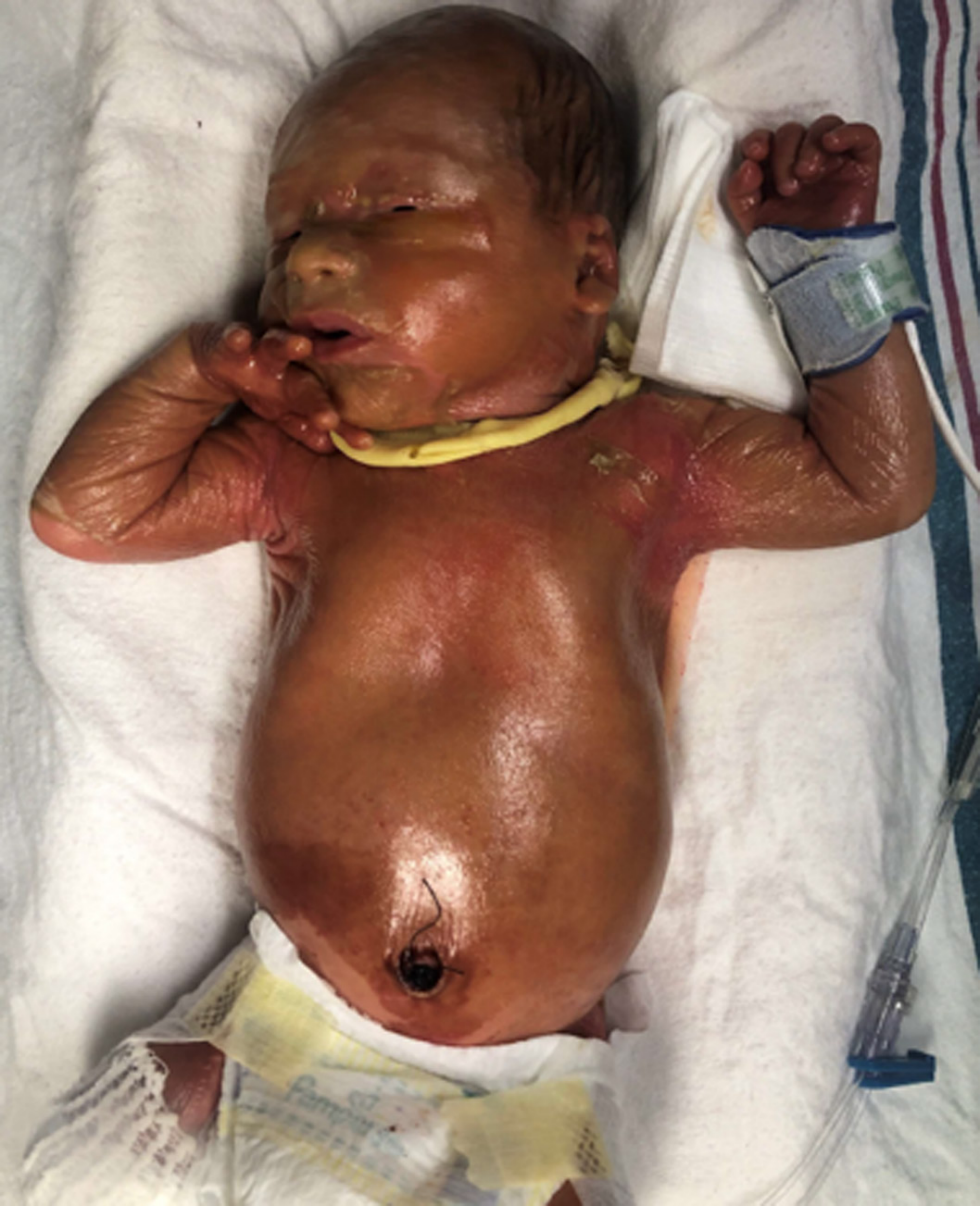
Collodion membrane just after birth.

A 34‐week gestational age male infant, born secondary to premature rupture of membranes presented with a collodion membrane at birth. Maternal history was significant only for Hepatitis C. There was no other contributory family history. Other than shortened femurs noted on prenatal ultrasound, the pregnancy was uncomplicated. The infant did not require significant resuscitation after delivery. On exam, the infant had diffusely shiny, tight parchment‐like skin; cracking skin in the neck and knee folds; and bilateral ectropion (Figure 1).

### 2.1 Investigations and treatment

The infant was transferred to a tertiary care center for pediatric subspecialty care. Genetics, dermatology, and ophthalmology were consulted. The infant was admitted to the NICU in a humidified isolette and placed on broad spectrum antibiotics until the blood culture was negative for 48 hours. Due to concern for skin integrity, a tunneled catheter was placed for venous access. The infant was weaned from the humidified isolette and intravenous fluids as his enteral feeds were increased. Congenital ichthyosis gene panel was performed, and the infant was heterozygous for the c.944 G > A mutation and the c.1025 G > T mutation on TGM1. He also had a c.4768 C > T mutation on the FLG gene. Due to concern for both ichthyosis vulgaris and lamellar ichthyosis, the dermatologist recommended use of bland emollients for skin care. The ophthalmologist recommended use of lubricating eye ointment until the infant was able to fully close his eyes. The infant's skin and eyes were monitored closely by ophthalmology and dermatology specialists. His course was prolonged by slow feeding due to prematurity. Hearing screen was not passed prior to discharge. The infant was gavage fed until he was able to take all enteral nutrition by bottle.

### 2.2 Outcome and follow‐up

**FIGURE 2:**
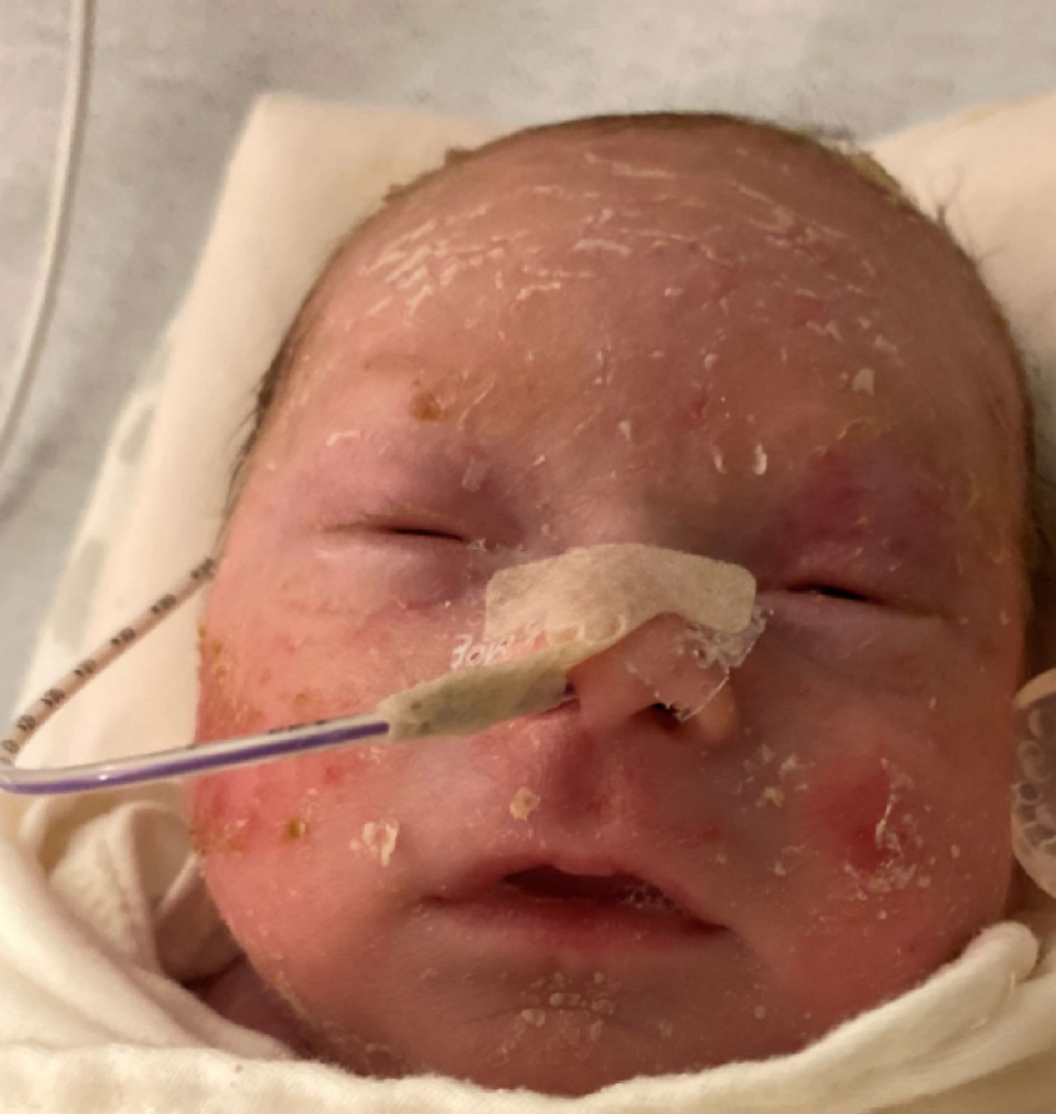
Slow desquamation and resolution of membrane. Both figures are photographs of actual patient during hospitalization.

As the collodion membrane healed, the infant developed brown plaques overlying the trunk and most prominent on the scalp (Figure 2). By 42 weeks corrected gestational age, the infant was discharged and no longer had collodion features or plaques but did have diffusely dry skin. Other than difficulty with using tape for nasogastric tube and avoiding use of chest leads, the infant's newborn course was uncomplicated from a dermatologic standpoint. The infant was followed closely in the dermatology clinic.

At 19 months of age, the patient had persistent eczematous patches in the axillae, trunk, and face with some scaly plaques on the scalp. These areas had markedly improved with the use of bland emollients when compared to initial presentation. The patient also has developmental delay, though it is unclear if this is related to the genetic mutations and has not previously been associated with either mutation. The infant does suffer from conductive hearing loss of the left ear, which has been rarely associated with congenital ichthyosis and ichthyosis vulgaris.^1^

## 3 DISCUSSION

An infant born with collodion membrane is rare, occurring in 1:50,000–1:100,000 live births.^2^ The membrane is the first presentation of autosomal recessive congenital ichthyosis (ARCI). ARCI occurs in 1:200,000–1: 300,000 births in the United States.^3^ The TGM1 gene encodes transglutaminase‐1.^3^ Variants in this gene are the most common causes of autosomal recessive congenital ichthyosis, which, in this case, likely led to the initial presentation of collodion membrane. Despite variants in a large number of genes being associated with congenital ichthyosis, a recently completed cohort study by Sun et al. concluded that collodion membrane at birth was significantly associated with mutations in TGM1 when compared to other genes.^4^ The infant in this case initially presented with a collodion membrane that following desquamation revealed thick, dark scales and plaques consistent with lamellar ichthyosis (LI).

The FLG gene encodes filament‐aggregating protein profilaggrin.^5^ Loss of function variants in this gene cause ichthyosis vulgaris, leading to long term dry, scaly skin.^5^ Ichthyosis vulgaris is common, occurring in 1:100–1:250 people, and generally presents outside of the newborn period. While both mutations seen in this patient are well‐documented individually, there is minimal documentation of patients with both mutations.

Few studies have evaluated hearing loss in ARCI, but a 2014 survey by *Huang et. al* showed that 28% of participants with ARCI had abnormal hearing results and 16% had hearing aids.^6^, ^7^ Ichthyosis vulgaris is linked to conductive hearing loss due to buildup of skin in the ear canal. There is no defined link between ACRI or ichthyosis vulgaris and developmental delay. There is, however, an association between developmental delay and sensorineural hearing loss associated with syndromic skin disorders.

In conclusion, while this infant with two mutations showed substantial improvement in skin condition over time, the persistence of eczematous patches and scaly plaques underscores the importance of continued follow‐up and monitoring. Furthermore, the presence of developmental delay and hearing impairment serves as a reminder of the need for comprehensive long‐term care and support for these patients.

## CONSENT

Written informed consent was obtained from the legal guardian to publish this report in accordance with the journal's patient consent policy.

## AUTHOR CONTRIBUTIONS

**Zackary Shearer:** Conceptualization; data curation; formal analysis; writing – original draft; writing – review and editing. **Gwenevere White:** Conceptualization; formal analysis; writing – review and editing. **John Zachary Steed:** Conceptualization; writing – review and editing. **Carla Brown:** Conceptualization; formal analysis; writing – review and editing. **Tara Venable:** Formal analysis; writing – review and editing. **Megan Baber:** Conceptualization; data curation; investigation; writing – review and editing.

## FUNDING INFORMATION

The authors recieved no financial support for the research, authorship, and publication of this article.

## CONFLICT OF INTEREST STATEMENT

All authors declare that they have no conflicts of interest.

## Data Availability

Data sharing not applicable to this article as no datasets were generated or analysed during the current study.
